# Supplementary material for: Involvement of Peripheral Monocytes with IL-1β in the Pathogenesis of West Syndrome
Source: J Clin Med. 2022 Jan 16;11(2):447. doi: 10.3390/jcm11020447 (PMC8779005; doi:10.3390/jcm11020447)
Supplement: Supplementary file 1 [file jcm-11-00447-s001.zip › jcm-1492393-supplementary.pdf]

Supplementary Materials

Table S1. Examination parameters for intracellular cytokines and plasma cytokine/chemokines.

1.1 Flow cytometry analysis

Molecule	Color	Clone	Isotype	Supplier
Lineage markers (Cell-surface staining)				
CD3	PerCP-Cy5.5	17A2	Rat IgG2b, κ	BioLegend
CD4	PE-Cy7	A161A1	Rat IgG2b, κ	
CD8	APC-Cy7	53-6.7	Rat IgG2a, κ	
CD19	APC-Cy7	6D5	Rat IgG2a, κ	
CD56	BV510	HCD56	Mouse IgG1, κ	
CD14	PE-Cy7	63D3	Mouse IgG1, κ	
Activation molecules (Cell-surface staining)				
CD25	FITC	M-A251	Mouse IgG1, κ	BioLegend
CD69	BV510	FN50	Mouse IgG1, κ	
CD86	PerCP-Cy5.5	BU63	Mouse IgG1, κ	
HLA-DR	PE	L243	Mouse IgG2a, κ	
CTLA-4	BV421	BNI3	Mouse IgG2a, κ	
Cytokines (Intracellular staining)				
IL-1β	AF647	JK1B-1	Mouse IgG1, κ	BioLegend
IL-1RA	FITC	CRM17	Mouse IgG1	eBioscience
IL-6	Pacific Blue	MQ2-13A5	Rat IgG1, κ	BioLegend
IL-10	BV421	JES3-9D7	Rat IgG1, κ	
IL-17	PE	9B10	Rat IgG2a, κ	
IFN-γ	APC	4S.B3	Mouse IgG1, κ	
TNF-α	BV510	MAb11	Mouse IgG1, κ	
Granzyme A	FITC	CB9	Mouse IgG1, κ	
FoxP3	AF647	150D	Mouse IgG1, κ	

1.2 Plasma cytokine/chemokine

**Th1-related cytokines**	**IFN-γ, TNF-α, IFN-γ-inducible protein 10 (IP-10)**
Th2-related cytokines	IL-4, IL-5, Eotaxin
Th17-related cytokines	IL-6, IL-8, IL-17, granulocyte colony-stimulating factor (G-CSF), and granulocyte macrophage colony-stimulating factor (GM-CSF)
Treg-related cytokine	IL-10

Others	IL-1β, IL-1RA, IL-12, IL-15, basic fibroblast growth factor (FGF-basic), Chemokine ligand (CCL) 2, MCP-1, CCL3 (MIP-1α), CCL4 (MIP-1β), platelet-derived growth factor-bb (PDGF-bb), regulated on activation, normal T expressed and secreted (RANTES), and vascular endothelial growth factor (VEGF)

Table S2. Comparison of intracellular cytokine levels between patients with West syndrome and the control participants.

		**1**	**2**	**3**	**4**	**5**	**6**	**7**	**8**	**9**	**10**	**11**	**12**	**13**
Monocytes	IL-1β	4.63	3.92	4.27	2.75	6.44	16.32	59.10	15.64	19.14	6.61	1.83	39.38	6.66
	IL-1RA	1.01	0.28	4.72	2.92	0.49	0.03	48.82	0.52	0.42	0.28	0.34	0.10	7.57
	IL-6	0.34	0.08	0.21	0.33	0.09	0.00	1.03	0.05	0.10	0.19	0.03	0.44	0.00
	TNF-α	1.21	0.78	0.35	0.56	0.08	0.00	2.30	0.47	0.54	0.50	0.27	15.15	0.10
**CD4$^+$ T cells**	IFN-γ	0.57	1.47	0.96	0.41	0.52	0.00	0.20	0.01	0.02	0.02	0.01	1.58	0.05
	GranzymeA	0.32	1.16	2.67	0.76	0.39	0.00	0.20	0.13	0.35	0.58	0.50	0.72	0.16
	IL-17	0.28	1.11	5.72	0.38	0.61	0.08	0.57	0.06	0.02	0.06	0.17	0.10	0.03
	IL-10	0.13	1.11	11.94	0.27	0.74	0.04	1.69	0.09	0.03	0.05	0.05	0.11	0.10
	IL-1β	0.13	0.21	0.30	0.02	0.47	0.35	0.34	0.34	0.85	0.82	0.17	0.04	0.20
	IL-1RA	0.72	0.33	0.43	0.26	0.10	3.96	6.19	1.02	2.01	1.13	6.20	0.04	1.01
	IL-6	0.26	0.07	0.38	0.25	0.01	0.00	0.08	0.02	0.02	0.01	0.01	0.07	0.00
	TNF-α	0.16	0.42	1.72	0.61	0.09	0.00	0.08	0.02	0.02	0.01	0.01	0.07	0.00
**CD8$^+$ T cells**	IFN-γ	20.77	15.33	7.34	5.81	0.21	0.01	1.15	0.12	0.12	0.06	0.00	0.64	0.04
	Granzyme A	0.51	0.40	6.76	1.85	0.65	0.01	16.47	0.19	0.27	0.93	0.29	0.17	0.15
	IL-17	0.08	0.36	5.03	0.10	0.18	0.03	0.30	0.01	0.01	0.00	0.07	0.02	0
	IL-10	0.09	0.31	5.46	0.08	0.20	0.01	2.36	0.02	0.02	0.03	0.04	0.04	0.01
	IL-1β	0.15	0.38	1.42	1.92	0.81	0.69	0.34	0.16	0.47	0.16	0.07	0.35	0.03
	IL-1RA	1.56	1.07	0.59	0.41	0.23	0.23	0.10	0.65	1.50	1.34	1.19	0.00	0.19
	IL-6	8.94	6.39	0.24	0.14	0.02	0.00	0.02	0.00	0.02	0.06	0.00	0.00	0.00
	TNF-α	0.02	0.04	2.66	1.16	0.04	0.00	0.16	0.00	0.07	0.06	0.00	0.03	0.00
**NKT-like cells**	IFN-γ	0.00	0.01	0.02	0.00	0.00	0.00	0.00	0.00	0.00	0.00	0.00	0.00	0.00
	Granzyme A	0.01	0.00	0.03	0.04	0.02	0.00	0.08	0.01	0.03	0.14	0.08	0.00	0.00
**NK cells**	IFN-γ	9.42	2.50	0.00	0.00	0.00	0.00	0.02	0.00	0.00	0.00	0.00	0.00	0.00
	Granzyme A	10.17	4.21	0.03	0.04	0.00	0.00	0.04	0.00	0.07	0.69	0.23	0.16	0.00
**B cells**	IFN-γ	0.01	0.00	0.02	0.04	2.00	0.07	0.04	0.01	0.00	0.01	0.00	0.00	0.24
	Granzyme A	0.82	0.21	0.15	1.89	1.24	0.00	4.54	0.16	0.31	0.46	0.62	0.02	0.15

Table S3. Plasma cytokine/chemokine concentrations of patients with West syndrome and control group participants.

	(pg/ml)	West syndrome group		Control group		*P*-value
**Th1**	IFN-γ	11.04	(9.37–76.01)	12.1	(8.1- 28.7)	1.00
	TNF-α	18.12	(4.26–63.83)	31.27	(8.08–44.94)	0.96
	IP-10	269.64	(157.97–435.02)	2917.3	(295.06–11456.77)	0.10
	IL-2	4.06	(2.69–7.86)	3.52	(0.91–4.13)	0.28
**Th 2**	IL-4	1.83	(1.48–2.72)	1.27	(0.7–2.18)	0.83
	IL-5	36.0	(18.3–62.0)	19.91	(0.6–40.78)	0.64
	Eotaxin	52.11	(36.38–65.24)	303.18	(64.28–806.55)	0.37
**Th 17**	IL-6	3.04	(2.58–17.03)	3.96	(3.78–14.08)	0.03
	IL-8	10.73	(7.13–1745.25)	6.79	(5.30–18.31)	0.76
	IL-17	4.51	(3.12–8.97)	6.65	(3.07–10.88)	1.00
	G-CSF	87.06	(70.79–804.06)	70.6	(20.02–103.79)	0.58
	GM-CSF	0.84	(0.32–2.28)	1.41	(0.4–6.055)	0.96
**Treg**	IL-10	6.12	(4.71–6.40)	7.68	(2.73–10.19)	0.15
**Others**	IL-1β	0.71	(0.53–11.53)	0.75	(0.53–1.11)	0.63
	IL-1RA	409.6	(377.29–1804.2)	293.93	(25.15–1070.17)	0.18
	IL-12	2.48	(1.89–4.56)	2.74	(2.09–4.00)	0.10
	IL-15	254.03	(184.36–291.81)	259.4	(0.00–309.37)	0.72
	FGF basic	30.45	(21.86–63.29)	29.8	(6.67–37.0)	1.00
	CCL2 (MCP-1)	119.64	(84.75–664.79)	49.1	(35.63–81.40)	0.64
	CCL3 (MIP-1α)	0.85	(0.78–58.9)	0.71	(0.4–0.87)	0.96
	CCL4 (MIP-1β)	146.76	(128.77–845.47)	125.3	(37.4–168.24)	0.70
	PDGF-BB	383.42	(283.77–813.76)	52.988	(222.63–1236.25)	0.64
	RANTES	8436.94	(5442.4–8603.1)	3257.77	(2020.7–8216.72)	0.72
	VEGF	441.64	(387.97–481.94)	438.75	(22.11–503.54)	0.18
